# Rapidly Evolving Cervical Dermatofibrosarcoma Protuberans With Deep Muscle Infiltration: A Rare and Aggressive Presentation

**DOI:** 10.7759/cureus.84596

**Published:** 2025-05-22

**Authors:** Oumaima Bouraqqadi, Sara Elloudi, Nawal Hammas, Nouha Boubekri, FatimaZahra Mernissi

**Affiliations:** 1 Dermatology Department, Hassan II University Hospital, Fez, MAR; 2 Biomedical and Translational Research Laboratory, Faculty of Medicine and Pharmacy, Sidi Mohammed Ben Abdellah University, Fez, MAR; 3 Radiology Department, Hassan II University Hospital, Fez, MAR

**Keywords:** darier–ferrand dermatofibrosarcoma, dermatofibrosarcoma protuberans, imatinib mesylate, imatinib therapy, oncodermatology

## Abstract

Dermatofibrosarcoma protuberans (DFSP) is a rare, low-grade sarcoma that typically presents as a slow-growing cutaneous nodule or plaque. We report a highly unusual case of DFSP involving the neck, which demonstrated rapid enlargement, necrosis, and deep muscle infiltration. A 45-year-old man presented with a large, bleeding cervical tumor that had evolved over two years. Imaging revealed extensive involvement of the neck and shoulder musculature without distant metastasis. Histopathologic evaluation confirmed the diagnosis of DFSP. Given the tumor’s inoperability, treatment with imatinib was initiated, resulting in marked tumor regression. This case highlights an atypical presentation of DFSP in terms of both location and clinical behavior. It underscores the importance of repeated histologic assessment in the face of rapid progression, the role of imaging in surgical planning, and the value of targeted therapy when surgical options are limited.

## Introduction

Dermatofibrosarcoma protuberans (DFSP) is a rare, locally aggressive soft tissue sarcoma that originates in the dermis and often extends into subcutaneous tissues, fascia, and, in advanced cases, muscle [1]. It typically presents as a slow-growing, firm, indurated plaque or nodule. However, its clinical appearance may vary, occasionally manifesting as a large tumor that may ulcerate and bleed as it progresses, leading to delays in diagnosis [2]. DFSP typically presents in young adults and is most commonly found on the trunk and proximal limbs, although involvement in the head and neck region is uncommon [1]. The tumor is known for its high recurrence rate after excision, particularly in cases with incomplete resection or tumors that have transformed [3]. DFSP includes several histological subtypes, including the classic form, the more aggressive fibrosarcomatous variant, and the pigmented Bednar tumor, each with distinct clinical implications [4]. The hallmark histopathological feature of DFSP is a spindle cell proliferation that is strongly positive for CD34, a diagnostic marker that helps distinguish it from other soft tissue sarcomas. A specific chromosomal translocation drives the tumor, t(17;22)(q22;q13), resulting in the COL1A1-PDGFB fusion gene, a finding that has diagnostic and therapeutic implications [1].

We present a case of cervical DFSP with unusual rapid growth, ulceration, and deep muscle infiltration, managed effectively with targeted therapy.

## Case presentation

A 45-year-old male with no significant medical history presented with a progressively enlarging, painful tumor on the left side of the neck. The lesion had begun two years prior as a small, painless 2 cm subcutaneous nodule. Approximately six months before consultation, a second erythematous, ulcerated, and hemorrhagic component developed, and the tumor rapidly increased in size. The patient denied any history of smoking, alcohol use, or prior illness, and baseline laboratory evaluations were within normal limits.

**Figure 1:**
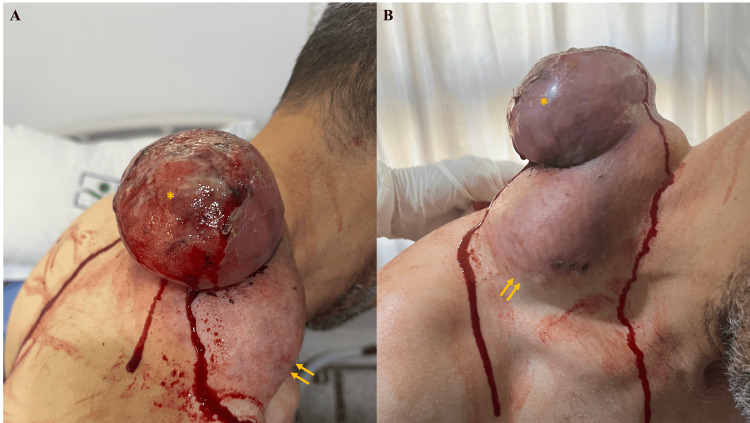
Clinical presentation of a well-defined subcutaneous tumor (~10 cm) in the basicervical region, with erythematous-violaceous overlying skin (yellow arrows). A second, superimposed angiomatous lesion, ulcerated and highly hemorrhagic, measuring approximately 6 cm is also visible (yellow asterisk). (A) Lateral view. (B) Frontal view

On examination, a well-defined subcutaneous tumor with regular contours was noted, measuring approximately 10 cm in its largest dimension. The tumor exhibited areas of firmness interspersed with softer regions, with an erythematous-violaceous overlying skin. Superimposed on the primary tumor was a second angiomatous lesion, ulcerated and highly hemorrhagic, measuring approximately 6 cm in its largest dimension (Figure 1).

**Figure 2:**
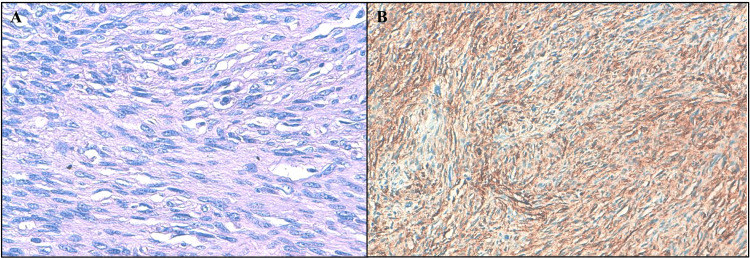
DFSP. (A) Tumor proliferation composed of spindle-shaped cells showing mild to moderate nuclear atypia (H&E, ×400). (B) Immunohistochemistry showing intense and diffuse positivity for CD34, consistent with DFSP H&E: hematoxylin and eosin, DFSP: dermatofibrosarcoma protuberans

The suspected diagnoses included DFSP with carcinomatous transformation, clear cell sarcoma, angiosarcoma, and Centrofollicular B-cell lymphoma. However, the biopsy revealed spindle cell proliferation with CD34 positivity (Figure 2), confirming the diagnosis of a DFSP.

A CT scan revealed a large tumoral process originating from the soft tissues of the left basi-cervical region and extending to the root of the ipsilateral limb. The mass was roughly oval, predominantly exophytic, with well-defined contours. It exhibited heterogeneous density and enhancement, with hypodense, non-enhancing areas corresponding to tumor necrosis. The tumor measured 92 x 95 mm in diameter.

**Figure 3:**
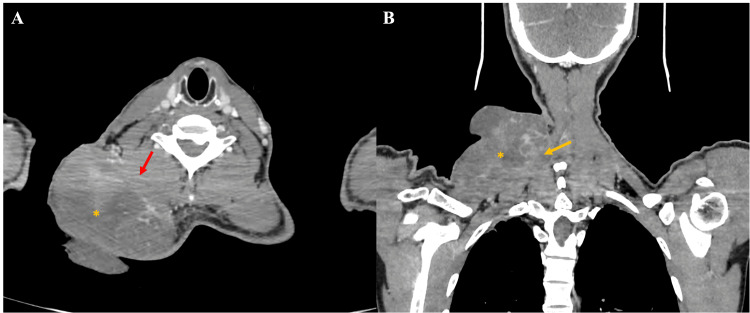
Helical CT scan in axial (A) and coronal (B) planes after iodinated contrast injection, showing an exophytic lesion (yellow asterisk) arising from the right posterior cervical soft tissues, with ill-defined, irregular margins. The lesion invades the right longus colli muscle (red arrow) and the ipsilateral trapezius muscle (yellow arrow) CT: computed tomography

The process infiltrated the left trapezius muscle anteriorly. Superiorly and inferiorly, it involved the levator scapulae muscle, the semispinalis muscle, the splenius capitis and cervical muscles, and the nuchal ligament (Figure 3). No lymph node involvement or signs of distant metastasis were observed.

**Figure 4:**
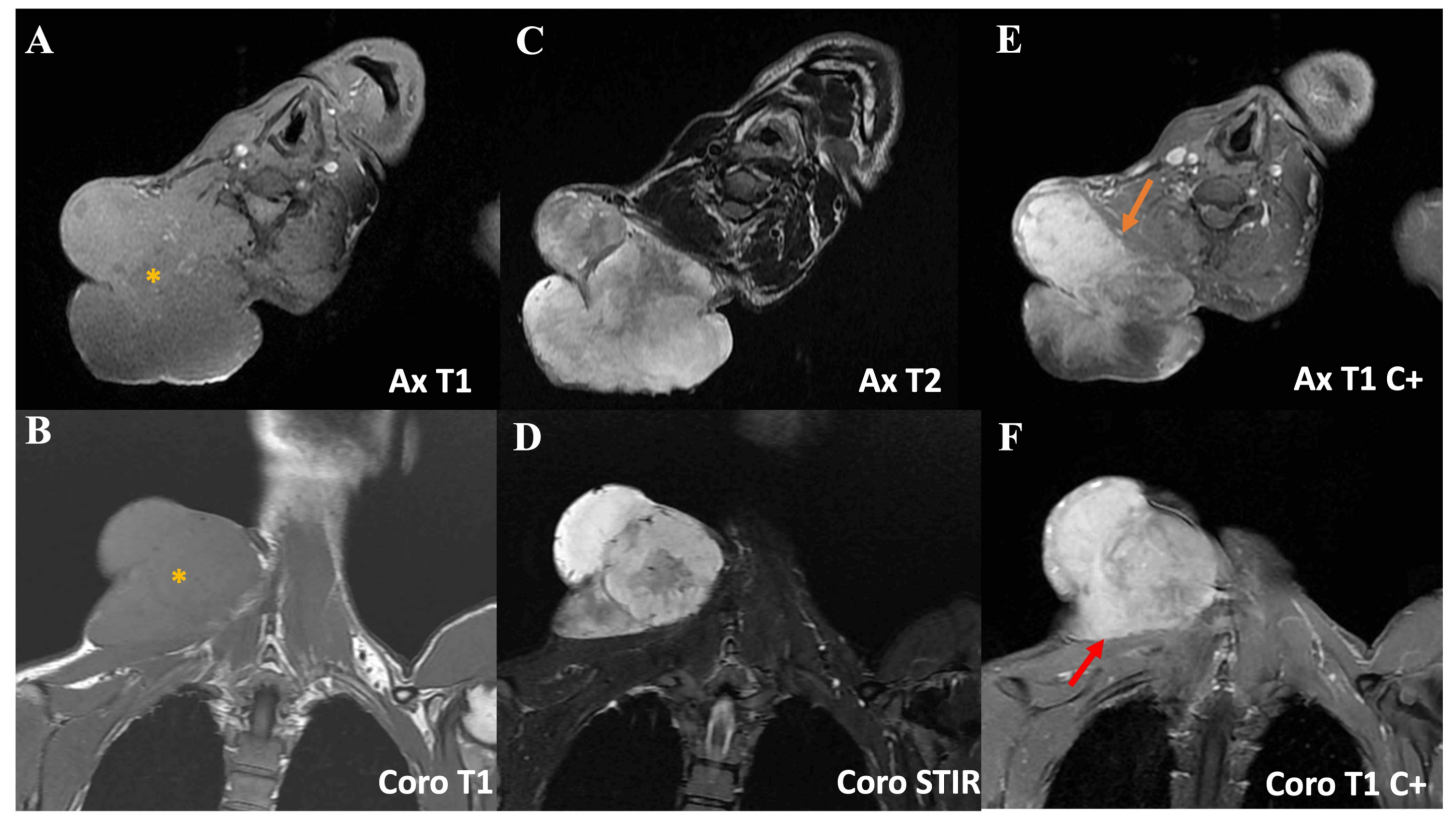
MRI showing a well-defined, lobulated mass (yellow asterisk) arising from the right basi-cervical soft tissues. The lesion appears isointense on T1-weighted images (A, B), hyperintense on T2/STIR sequences (C, D), and shows heterogeneous enhancement after contrast (E, F). It invades the ipsilateral longus colli (orange arrow) and trapezius muscles (red arrow) MRI: magnetic resonance imaging

An MRI was performed (Figure 4), confirming the same findings, with no evidence of vascular infiltration.

**Figure 5:**
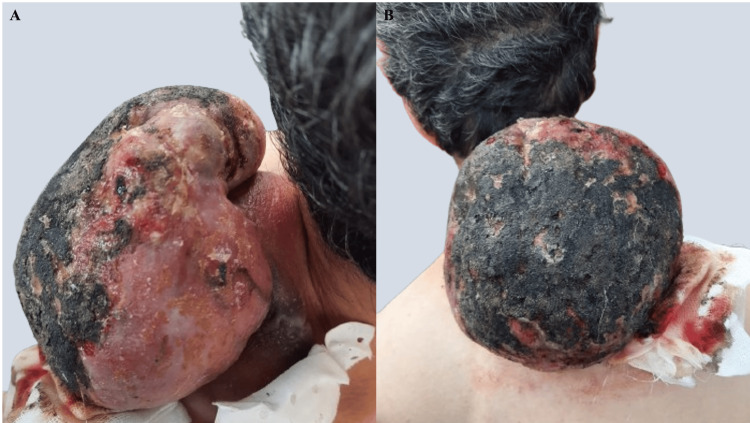
Marked increase in the size of the angiomatous lesion one month after the initial consultation. The lesion became largely necrotic and measured approximately 15 cm in diameter. (A) Lateral view. (B) Posterior view

Approximately one month after the initial consultation, the patient reported an increase in the size of the angiomatous lesion, which became largely necrotic and measured approximately 15 cm in diameter (Figure 5).

Given the rapid progression and to further clarify the diagnosis, the patient underwent multiple cutaneous and echo-guided biopsies to rule out carcinomatous transformation or the coexistence of an angiosarcoma. However, all biopsy results consistently confirmed DFSP.

**Figure 6:**
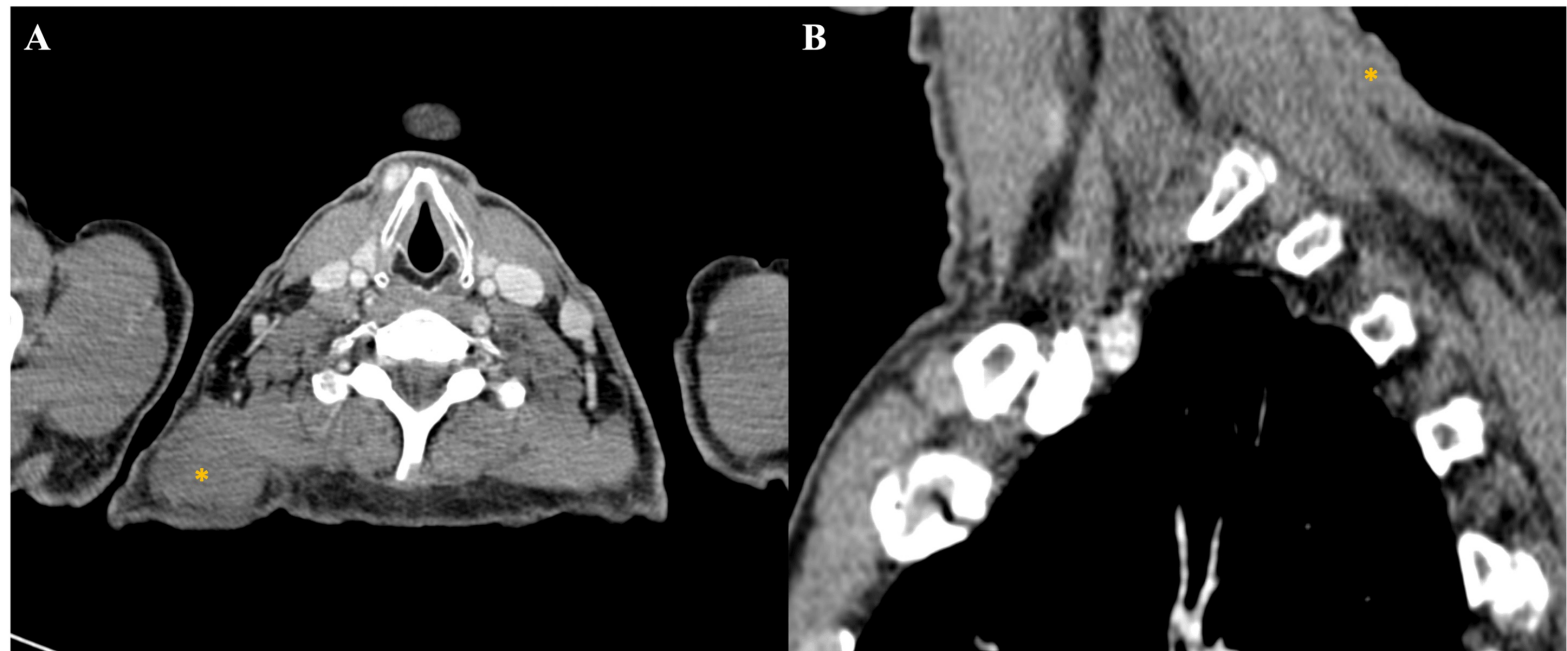
Helical CT acquisition in axial (A) and sagittal (B) planes showing significant reduction in the size of the mass (yellow asterisk) arising from the right basi-cervical soft tissues, still in close contact with the ipsilateral trapezius muscle CT: computed tomography

**Figure 7:**
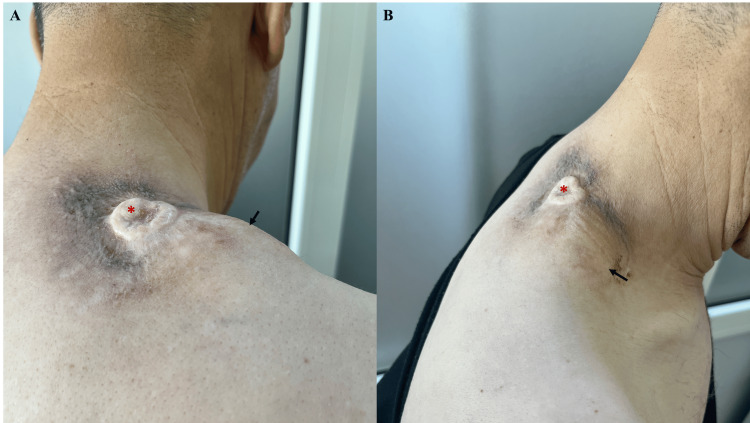
Marked reduction of the subcutaneous tumor four months after initiation of imatinib therapy (arrows), with complete regression of the angiomatous component (asterisk). (A) Posterior view. (B) Lateral view

Given the locally advanced nature of the tumor, involving multiple muscles of the neck and shoulder, the case was discussed in a multidisciplinary tumor board. Given the extent of infiltration, the patient was deemed inoperable. A decision was made to initiate treatment with imatinib at a dosage of 800 mg/day. After four months of therapy, a follow-up CT scan showed a significant reduction in the size of the tumoral mass (Figure 6), now measuring 68 x 42 x 18 mm in the soft tissues of the right basi-cervical region (Figure 7). Despite this response, the tumor remains unresectable.

The patient continues to receive imatinib treatment and remains under close monitoring with quarterly follow-up to assess for any signs of progression or recurrence.

## Discussion

DFSP is a rare, slow-growing soft tissue sarcoma typically arising in the dermis or subcutis, with an incidence estimated at approximately six cases per million annually [1,5]. It most commonly affects the trunk but can occur anywhere on the body. The age distribution is broad, peaking between 20 and 59 years, with a slight male predominance [6]. Epidemiological data have shown that DFSP incidence is higher among Black populations compared to White populations, and tumors located in the head and neck region, such as in our patient, are less frequent but tend to be associated with more aggressive behavior and worse prognosis [1,4,5].

Early DFSP is often asymptomatic, contributing to delayed diagnosis [7]. This delay was evident in our case, where the patient had a two-year history of a painless, bleeding neck mass before presentation. The tumor’s clinical progression from a small nodule to a large, ulcerated, and hemorrhagic mass is consistent with the natural history of DFSP. However, the extensive involvement of neck and shoulder muscles, including the trapezius, levator scapulae, and splenius muscles, represents a particularly aggressive local invasion, underscoring the challenges posed by DFSP in anatomically complex regions like the head and neck [8].

Imaging modalities such as CT and MRI are essential in evaluating the local extent of disease, helping guide management decisions. In our patient, imaging confirmed significant local invasion without evidence of distant metastasis, a finding in line with the typical low metastatic potential but locally aggressive nature of DFSP [4,5].

Surgical excision with clear margins remains the standard of care for DFSP; however, when tumors are deemed unresectable due to size or infiltration of critical structures, systemic therapy becomes necessary [4,5]. Imatinib, a tyrosine kinase inhibitor, has shown promising results in treating advanced DFSP, particularly in cases with metastatic disease or inoperable cases [9]. The drug targets the platelet-derived growth factor receptor, which is often overexpressed in DFSP, leading to tumor growth [9]. In this case, the patient’s tumor showed significant regression after four months of imatinib therapy, demonstrating the potential of targeted therapy in controlling disease progression and reducing tumor size. The patient remains on imatinib with close monitoring, as recurrence is common in DFSP even after initial treatment success [3,9].

The role of imatinib in DFSP, particularly in locally advanced or metastatic cases, has been well documented in the literature [1,9]. Several studies have reported favorable outcomes with imatinib in unresectable cases or have recurred after surgery [9,10]. However, the optimal duration of treatment and long-term outcomes remain areas of ongoing research. This case adds to the growing body of evidence supporting imatinib as a viable treatment option for advanced DFSP, offering hope for patients not candidates for surgical resection.

Histopathologically, DFSP is characterized by spindle cell proliferation with a storiform pattern and diffuse CD34 positivity, which was confirmed in our biopsy specimens [4]. Molecular testing for the characteristic fusion gene was not performed due to resource limitations, a limitation we acknowledge. Nevertheless, the clinical, histological, and immunohistochemical findings strongly support the diagnosis.

## Conclusions

This case underscores the diagnostic and therapeutic challenges posed by DFSP in an uncommon cervical location with extensive invasion of multiple neck and shoulder muscles. The aggressive local behavior observed highlights the importance of early recognition and thorough histopathologic evaluation. Although molecular testing for the COL1A1-PDGFB fusion gene was not performed due to resource limitations, the diagnosis was confirmed by characteristic histology and immunohistochemistry. Given the tumor’s inoperability, targeted therapy with imatinib led to significant tumor regression and disease control. This case emphasizes the critical role of multidisciplinary collaboration and close clinical monitoring in managing advanced DFSP to optimize patient outcomes.
